# Lysophosphatidylcholine plays critical role in allergic airway disease manifestation

**DOI:** 10.1038/srep27430

**Published:** 2016-06-10

**Authors:** Preeti Bansal, Shailendera Nath Gaur, Naveen Arora

**Affiliations:** 1 Allergy and Immunology Section, CSIR-Institute of Genomics and Integrative Biology, Delhi; 2 Department of Biotechnology, University of Pune, Ganeshkhind, Pune 411 007, India; 3 Department of Pulmonary Medicine,V.P.Chest Institue, Delhi University, Delhi

## Abstract

Phospholipase A2 (sPLA2), pivotal for allergic and inflammatory response, hydrolyses phosphatidylcholine (PC) to lysophosphatidylcholine (LPC). In present study, the role of LPC in allergic airway disease manifestation was studied using mouse model. Balb/c mice were immunized using cockroach extract (CE) and LPC release was blocked by sPLA2 inhibitor. Airway hyperresponse (AHR), lung-histology, total and differential leukocyte count (TLC&DLC), Th2 type cytokines, sPLA2 activity and LPC levels in bronchoalveolar lavage fluid (BALF) were measured. Exogenous LPC was given to the mice with or without CE sensitization, to demonstrate its role in allergic airway disease manifestation. Anti-CD1d antibody was given to study the involvement of natural killer T (NKT) cells in LPC induced response. AHR, lung-inflammation, TLC, DLC, Th2 type cytokines, sPLA2 activity and LPC levels were increased on CE challenge. sPLA2 activity and LPC release was blocked by sPLA2-inhibitor, which decreased AHR, and inflammatory parameters. Exogenous LPC with or without CE sensitization increased above parameters. CE challenge or LPC exposure increased LY49C^+^TCRβ^+^ NKT cells in BALF and spleen, which was reduced by anti-CD1d antibody, accompanied with reduction in AHR and allergic airway inflammation parameters. Conclusively, LPC induces allergic airway disease manifestation and it does so probably *via* CD1d-restricted LY49C^+^TCRβ^+^ NKT cells.

Lysophosphatidylcholine (LPC) is a lipid whose level increases in plasma and bronchoalveolar lavage fluid (BALF) of asthma and rhinitis patients^1^,^2^. LPC is produced from phospholipids by the action of phospholipase A2 (PLA2)^3^. Depending upon the location, PLA2 may be cytoplasmic (cPLA2) or secretory (sPLA2). Increased level of sPLA2 has been observed in the bronchoalveolar lavage of asthmatics^4^.

Allergen challenge induces PLA2 (sPLA2) secretion by various airways cells^1^,^4^,^5^. sPLA2 is a pivotal enzyme involved in allergic response and the inflammatory-asthmatic response^6^. The link between sPLA2 downstream pathway and allergic manifestation is yet to be studied.

Lung mast cells store sPLA2 in its granules. Cross-linking of IgE on allergen challenge induces degranulation of mast cells releasing sPLA2 in the extracellular fluid in early phase of allergic reaction^4^. Beside mast cells, alveolar macrophages and neutrophils also secrete sPLA2^7^,^8^. sPLA2 hydrolyses phospholipids of the cell membrane at the sn-2 position of ester bond resulting in LPC and free fatty acid or arachidonic acid^3^. The latter is a precursor molecule for various eicosanoids including prostaglandins and leukotrienes^9^ and is not involved in initiation of allergic response, but only amplifies the inflammation^10^.

Role of LPC has been seen in desensitization of β-adrenergic receptor by Ca^2+^ sensitization in tracheal smooth muscle cells^11^. It involves in eosinophils infiltration and bronchoconstriction^12^,^13^. These reports indicated the link of LPC and allergic airway disease like asthma. In present study, the secretion of sPLA2 and release of LPC was analysed in mice challenged with cockroach extract (CE). Exogenous LPC was given to mice to study its involvement in allergic cascade.

Various studies have indicated that natural killer T (NKT) cells involve in initiation of the allergic response^14^,^15^. A study by Lisbonne *et al*. has indicated the formation of an endogenous lipid on allergen challenge may involve in activation of NKT cells^15^. NKT cells are CD1d restricted cells, which get activated by a lipid bound to CD1d of the antigen presenting cells (APCs). In present study, it was hypothesized that LPC *via* CD1d may activate NKT cells triggering the airway allergic cascade. This hypothesis was tested by administration of monoclonal antibody (mAb) against CD1d before exposure to LPC or challenge with CE.

## Results

### Airway hyperresponse (AHR), lung inflammation, and Th2 type cytokines increase on challenge with CE

**Figure 1:**
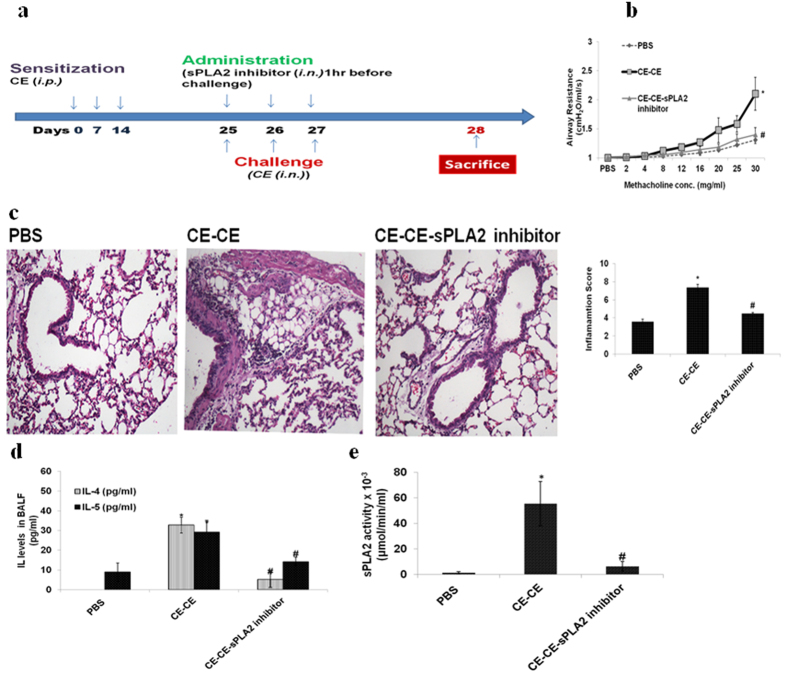
AHR, lung inflammation, Th2 type cytokines and sPLA2 activity were increased in CE challenged mice and were reduced when administered with sPLA2 inhibitor. (**a**) Immunization protocol. (**b**) Airway Resistance (**c**) H&E stained lung sections and inflammation score (**d**) IL-4 and IL-5 (**e**) sPLA2 activity. Vehicle control: *PBS*; mice sensitized and challenged with cockroach extract: *CE-CE*; and pre-administered with sPLA2 inhibitor: *CE-CE-sPLA2 inhibitor*. Data represent the means  ± SEM of values from 4 mice. *p < 0.05 versus *PBS;*^#^p < 0.05 versus *CE-CE*.

To mimic natural challenge with allergen mice were exposed to CE (Fig. 1a). It was observed that the AHR was increased significantly in CE challenged mice as compared to Phosphate buffer Saline (PBS) control mice (p < 0.05) (Fig. 1b). There was significant increase in Total leukocyte Count (TLC) (p < 0.05) and Differential leukocyte Count (DLC) (p < 0.05) in CE challenged mice (Supplementary Fig. 1). Similarly, lungs were significantly inflamed in CE immunized mice as demonstrated by lung histology and inflammation score (p < 0.05) (Fig. 1c). Th2 type cytokines IL-4 and IL-5 were significantly increased in BALF of CE challenged mice (p < 0.05) (Fig. 1d).

### LPC level increases in proportion to sPLA2 activity in BALF of CE challenged mice

sPLA2 activity showed a significant increase in CE challenged mice as compared to PBS control mice (p < 0.05) (Fig. 1e). LPC16:0 level was also significantly increased in BALF of CE challenged mice as compared to PBS control mice (p < 0.05) (Table 1). LPC18:0 and LPC18:1 was not showing any significant change among the groups (Data not shown).

### sPLA2 inhibitor decreases AHR, inflammation and Th2 type cytokines up to the level of control mice

Pre-administration of sPLA2 inhibitor (Fig. 1a) showed significant decrease in AHR in CE challenged mice as compared to dimethylsulfoxide (DMSO) administered mice (p < 0.05). The reduction was almost to the level of PBS control mice (Fig. 1b). Similarly, inflammation as demonstrated by TLC, DLC (Supplementary Fig. 1) and lung histology and inflammation score was decreased significantly (p < 0.05) (Fig. 1c). In similar way, Th2 type cytokines were decreased significantly (p < 0.05) to the level of PBS control mice (Fig. 1d).

### Inhibitor decreases sPLA2 activity and LPC level in CE challenged mice

It was observed that sPLA2 activity was decreased by the inhibitor (Fig. 1e). As a consequence, LPC synthesis was also hindered (Table 1).

**Figure 2:**
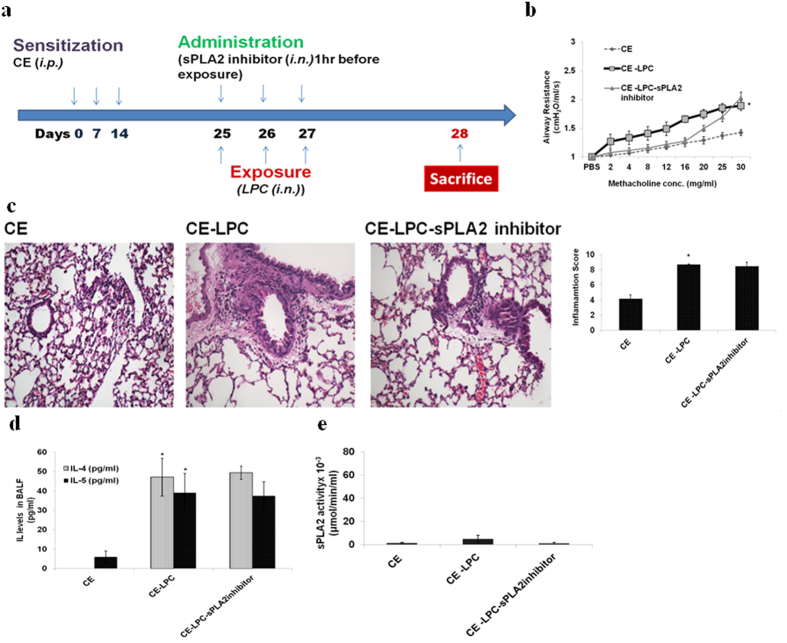
LPC exposure increases AHR, lung inflammation, Th2 type cytokines with no change in sPLA2 activity. (**a**) Immunization protocol. (**b**) Airway Resistance (**c**) H&E stained lung sections and inflammation score (**d**) IL-4 and IL-5. (**e**) sPLA2 activity. Mice sensitized with cockroach extract: *CE*; and exposed to LPC: *CE-LPC*; and pre-treated with sPLA2 inhibitor: *CE-LPC-sPLA2inhibitor*. Data represent the means ± SEM of values from 4 mice. *p < 0.05 versus *CE*.

Above results indicated the direct co-relation of LPC concentration and allergic airway disease parameters. To confirm the role of LPC in allergic cascade trigger, mice were directly exposed to LPC after sensitization to CE (Fig. 2a). CE sensitized mice without any challenge were taken as control group.

### LPC exposure increases AHR, inflammation and Th2 type cytokines

LPC exposure increased AHR significantly as compared to control mice (p < 0.05) (Fig. 2b). Inflammation as demonstrated by TLC, DLC, (Supplementary Fig. 2) lung histology and inflammation score (Fig. 2c) was significantly high in LPC exposed mice (p < 0.05). IL-4 and IL-5 were significantly increased in LPC exposed mice (p < 0.05) (Fig. 2d). No effect was observed in these parameters, when LPC exposed mice were pre-administered with sPLA2 inhibitor.

### sPLA2 activity was less in mice exposed to LPC

CE sensitization and LPC exposure did not show any significant sPLA2 activity in BALF (Fig. 2e) and hence no release of LPC *in vivo*. The effect observed was due to exogenous LPC. Therefore, no effect of sPLA2 inhibitor was observed in LPC exposed mice.

### LPC exposure without CE sensitization increases AHR, inflammation and Th2 type cytokines

**Figure 3:**
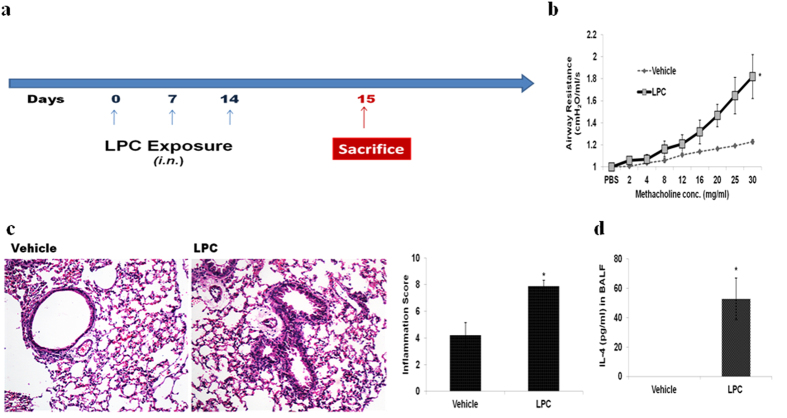
LPC exposure without CE sensitization increases AHR, lung inflammation and IL-4. (**a**) Immunization protocol. (**b**) Airway resistance (**c**) Lung sections stained with H&E and inflammation score. (**d**) IL-4 in BALF. Mice exposed to 5%DMSO: *Vehicle*; to LPC: *LPC*. Data represent the means ± SEM of values from 4 mice. *p < 0.05 versus *vehicle*.

To further confirm the involvement of LPC in allergic cascade, we directly exposed the mice to exogenous LPC, without CE sensitization (Fig. 3a). We found a significant increase in AHR (p < 0.05) in LPC exposed mice as compared to vehicle control mice (Fig. 3b). Airway inflammation as demonstrated by TLC (p < 0.05), DLC (p < 0.05) (Supplementary Fig. 3), lung histology and inflammation score (p < 0.05) (Fig. 3c) and cytokine IL-4 level (p < 0.05) (Fig. 3d) were increased in LPC exposed mice.

**Figure 4:**
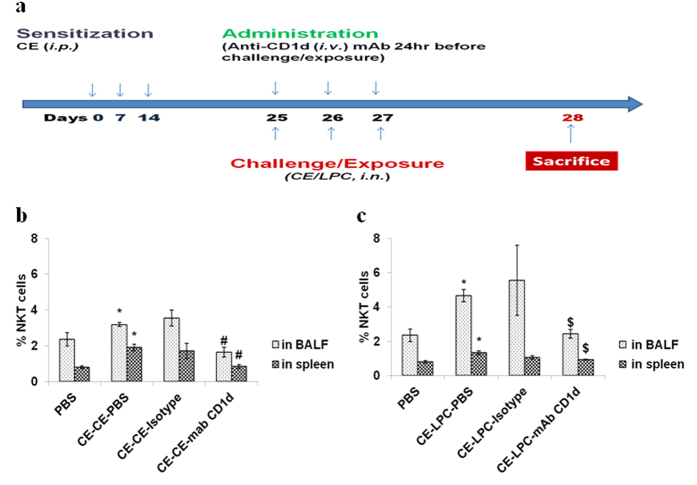
CE challenge increases and CD1d blockage decreases NKT cells in BALF and spleen of mice. (**a**) Immunization protocol (**b,c**) NKT cells in BALF and spleen. NKT cells are expressed as the percentage NKT cells, i.e. % proportion of the NKT cells out of the total cells in BALF or spleen. In part b, vehicle control: *PBS*; mice sensitized and challenged with cockroach extract and pre-administered with PBS: *CE-CE-PBS*; with isotype: *CE-CE-Isotype;* or with anti-CD1d monoclonal antibody (mAb): *CE-CE-mAb CD1d*. In part c, vehicle control: *PBS*; Mice sensitized with cockroach extract and exposed to LPC and pre-administered with PBS: *CE-LPC-PBS*; or with isotype: *CE-LPC-Isotype*; or with anti-CD1d mAb: *CE-LPC-mAB CD1d.* Data represent the means ± SEM of values from 4 mice. *p < 0.05 versus *PBS*; ^#^p < 0.05 versus *CE-CE-PBS*, ^$^p < 0.05 versus *CE-LPC-PBS*.

Further we studied that how the LPC is leading to inflammatory manifestation. NKT cells are known to involve in initiation of allergic response and are CD1d restricted. LPC *via* CD1d might be involving in NKT cell activation^14^,^15^. Hence, we used anti-CD1d mAb to block NKT cells activation *in vivo* (Fig. 4a). For comparison we also used CE challenged mice model.

### CE challenge or LPC exposure increases and CD1d blockage decreases the NKT cells in BALF and spleen

LY49C^+^TCRβ^+^ NKT cells were increased significantly in BALF of CE challenged or LPC exposed mice as compared to PBS control mice (p < 0.05) (Fig. 4b,c). Similar to pattern observed in BALF, NKT cells were also increased significantly in spleen of CE challenged or LPC exposed mice (p < 0.05) (Fig. 4b,c). Administration of blocking antibody to CD1d decreased LY49C^+^TCRβ^+^ NKT cells significantly (p < 0.05) in BALF of CE challenged (Fig. 4b) or LPC exposed mice (Fig. 4c). Similarly, NKT cells were decreased significantly (p < 0.05) in spleen of CE challenged mice (Fig. 4b) or LPC exposed mice on administration of the antibody (Fig. 4c). There was no significant change in NKT cells on administration of isotype antibody.

### CD1d blockage decreases AHR, airway inflammation and Th2 type cytokines

**Figure 5:**
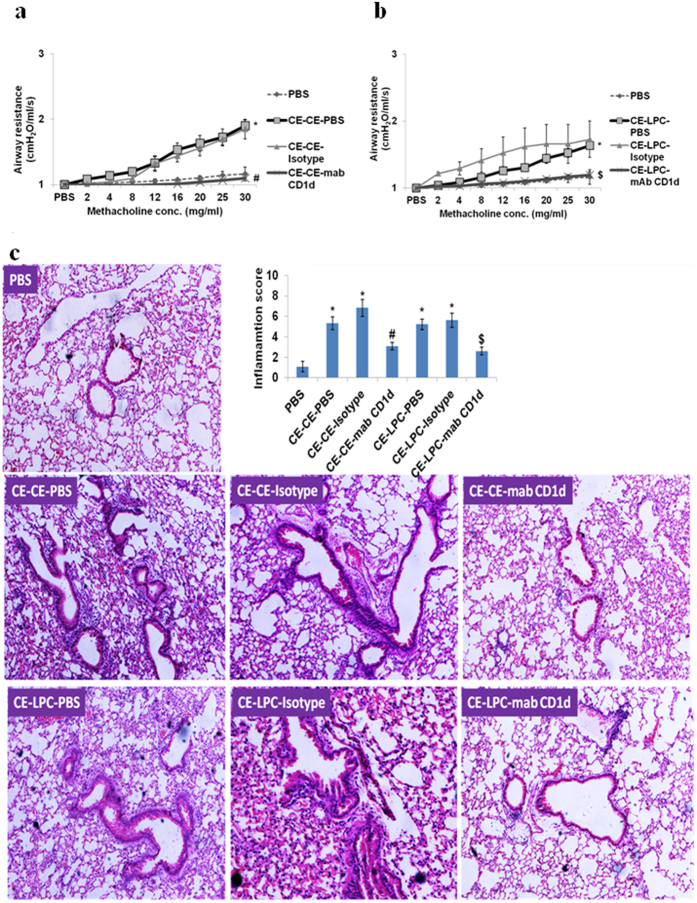
CD1d blockage before CE challenge or LPC exposure decreases AHR and lung inflammation. (**a,b**) Airway resistance (**c)** H&E stained lung sections and inflammation score. In part a&c, vehicle control: *PBS*; mice sensitized and challenged with cockroach extract and pre-administered with PBS: *CE-CE-PBS*; with isotype: *CE-CE-Isotype;* or with anti-CD1d monoclonal antibody (mAb): *CE-CE-mAb CD1d*. In part b&c, vehicle control: *PBS*; mice sensitized with cockroach extract and exposed to LPC and pre-administered with PBS: *CE-LPC-PBS*; or with isotype: *CE-LPC-Isotype*; or with anti-CD1d mAb: *CE-LPC-mAB CD1d.* Data represent the means ± SEM of values from 4 mice. *p < 0.05 versus *PBS*; ^#^p < 0.05 versus *CE-CE-PBS*, ^$^p < 0.05 versus *CE-LPC-PBS*.

CD1d blockage in CE challenged or LPC exposed mice decreased AHR (Fig. 5a,b), TLC, eosinophils, neutrophils (Supplementary Fig. 4a,b), and lung inflammation significantly (p < 0.05) (Fig. 5c). Similarly, IL-4, IL-5 and IL-13 levels in BALF were decreased significantly (p < 0.05) (Table 2).

Above results demonstrated that LPC triggers for the manifestation of these parameters probably by activating NKT cells *via* CD1d.

### Blockage of CD1d reduces oxidative stress in CE challenged mice, with no reduction in LPC exposed mice

**Figure 6:**
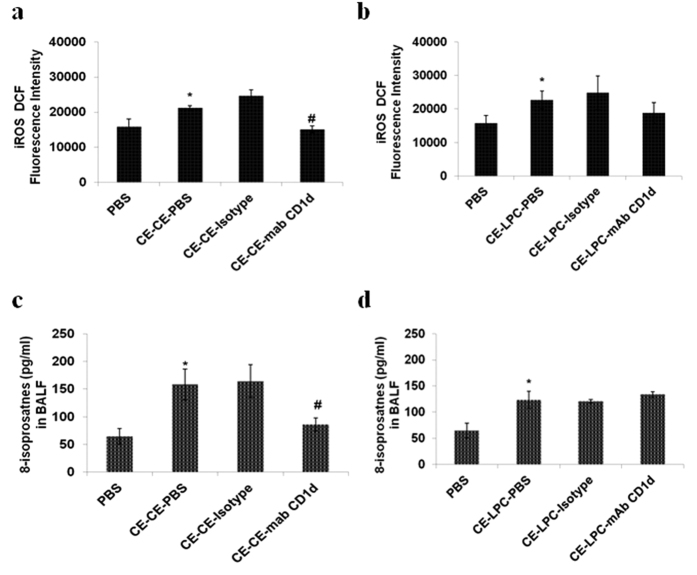
Blockage of CD1d reduces the iROS and 8-isoprostanes level in CE challenged mice but not in LPC exposed mice. (**a,b)** iROS levels in lung homogenate (**c,d)** 8-isoprostanes levels in BALF. In part a&c, vehicle control: *PBS*; mice sensitized and challenged with cockroach extract and pre-administered with PBS: *CE-CE-PBS*; with isotype: *CE-CE-Isotype*; or with anti-CD1d monoclonal antibody (mAb): *CE-CE-mAb CD1d*. In part b&d, vehicle control: *PBS*; mice sensitized with cockroach extract and exposed to LPC and pre-administered with PBS: *CE-LPC-PBS*; or with isotype: *CE-LPC-Isotype*; or with anti-CD1d mAb: *CE-LPC-mAB CD1d.* Data represent the means ± SEM of values from 4 mice. *p < 0.05 versus *PBS*; ^#^p < 0.05 versus *CE-CE-PBS*, ^$^p < 0.05 versus *CE-LPC-PBS*.

CE challenge or LPC exposure led to significant increase in intracellular reactive oxygen species (iROS) (p < 0.05) (Fig. 6a,b) and 8-isoprostanes levels (p < 0.05) (Fig. 6c,d) as compared to PBS control mice. Administration of anti-CD1d mAb in CE challenged mice decreased iROS or 8-isoprostanes levels significantly (p < 0.05) (Fig. 6a,c). However, its administration in LPC exposed mice did not cause any change in these parameters (Fig. 6b,d).

## Discussion

Allergen challenge with Timothy grass, dust mite, etc. leads to the secretion of sPLA2^1^,^4^,^5^. The increased activity of sPLA2 has been seen in BALF and serum of asthma patients^16^,^17^. Shoseyov *et al*. have shown that sPLA2 is critical for an allergic response^6^. The link between sPLA2 downstream pathway and allergic manifestation is yet to be studied.

sPLA2 after secretion acts over phospholipids of cell membranes and leads to the formation of LPC and fatty acid including arachidonic acid which is the precursor molecule for various eicosanoids^3^,^9^. Calder *et al*. reported that arachidonic acid is not critical for triggering the allergic cascade^10^. LPC may play an important role in allergic disease. In present study, the role of LPC in the allergic manifestation was studied using mice model. sPLA2 inhibitor given 1hr before challenge attenuated the CE-induced allergic response indicating its importance for the allergic disease.

sPLA2 inhibitor administration before CE challenge reduces the airway hyper response to the level of control mice. LPC exposure leads to a significant increase in the response as compared to control mice demonstrating the role of LPC in AHR. This observation is consistent with the earlier studies^6^.

The present study showed that LPC is involved in Th2 type cytokines secretion, as seen by increase in IL-4 and IL-5 in mice directly exposed to LPC. It was observed that sPLA2 inhibitor administration in CE immunized mice not only inhibited sPLA2 activity, but, also Th2 type cytokines secretion. This is the first *in vivo* report regarding LPC involvement in Th2 type cytokines secretion in allergic airway disease.

Earlier reports have shown that LPC induces expression of various adhesion molecules and chemokines in vascular endothelium^18^,^19^. It increases the permeability of the endothelium^20^ and probably has a role in inflammation. In the present study, we observed a significant increase in total cell count including eosinophils, neutrophils, lymphocytes, monocytes, and platelets, as well as lung inflammation on LPC exposure probably, *via* increased adhesion molecules and chemokines. The LPC induced response was further confirmed by exposure to LPC without CE sensitization.

Further, it was observed that CE challenge or LPC exposure significantly increases LY49C^+^TCRβ^+^ NKT cells in BALF indicating LPC involvement in activation of NKT cells. To study the pathway involved in activation of NKT cells, anti-CD1d mAb was administered before the CE challenge or LPC exposure. In earlier studies, anti-CD1d monoclonal antibody has been used *in vivo* to block NKT cells activation^15^,^21^,^22^. In the present study too, NKT cell activation was blocked using anti-CD1d mAb. mAb administration in CE challenged or LPC exposed mice, reduced NKT cells activation. Increased number of NKT cells in spleen also indicated the systemic effect of LPC. Many studies have reported the increased NKT cells in the lungs of asthma patients^23^,^24^,^25^,^26^,^27^. In the present study, anti-CD1d antibody administration in CE challenged or LPC exposed mice decreased AHR significantly, indicating LPC induced NKTs are directly involved in AHR. Similarly, decrease in Th2 type cytokines with the antibody administration, demonstrates the involvement of LPC-induced NKT cells in Th2 type cytokines secretion. Lisbone *et al*. reported that anti-CD1d antibody administration blocked AHR and the ability of NKT cells to secrete Th2 type cytokines^15^. Many studies have reported that NKT cells have high potential of secreting Th2 type cytokines like IL-4^15^,^28^.

Further significant decrease in airway inflammation of CE challenged or LPC exposed mice by anti-CD1d antibody administration indicates the LPC induced NKT cells involvement in lung inflammation. A significant decrease in eosinophils has been reported in previous studies in the absence of NKT cells^14^,^29^.

There was a significant decrease in oxidative stress of CE challenged mice but, not in LPC exposed mice on administration of antibody. It might be due to high level of exogenous LPC than endogenous level, activating other pathways and hence oxidative stress was not inhibited by blocking CD1d.

Various studies have reported the involvement of NKT cells in allergic diseases but, the trigger for the activation of these cells, in allergic manifestation is not known. Although, some studies indicated that LPC might activate NKT cells^30^,^31^, but, the present study is the first *in vivo* report regarding activation of NKT cells by LPC in case of allergic airway disease. The down regulation of allergic airway disease parameters was due to blockage of CD1d-NKT signalling and not due to depletion of APCs^21^,^22^.

Study demonstrates that allergen challenge leads to secretion of PLA2 in BALF; this acts on the phospholipids to release LPC and fatty acid. LPC *via* CD1d of APCs activates LY49C^+^TCRβ^+^ NKT cells and leads to AHR and IL-4, IL-5 and IL-13 secretion. LPC induces expression of various adhesion molecules on the vascular endothelium and increases permeability, thus increases inflammation in the lungs^19^,^20^ by inflammatory cells recruitment. The cells produce iROS, which induces formation of 8-isoprostanes contributing in oxidative stress. In conclusion, LPC induces allergic airway disease manifestation and it does so probably *via* CD1d-restricted LY49C^+^TCRβ^+^ NKT cells.

## Methods

The study design was approved by the animal ethics committee of CSIR-Institute of Genomics and Integrative Biology. All the experiments were performed according to the guidelines of committee for the purpose of control and supervision of experiments on animals (CPCSEA), Ministry of Environment, Forests and Climate change, govt. of India. The experiments were carried out twice independently and data presented here belongs to one of the two experiments with similar results.

### Preparation of cockroach extract (CE)

The whole body cockroach extract was prepared by method described earlier^32^.

### Mice

Female BALB/c mice (4–6 weeks old), weighing 20–25 g, were obtained from National Institute of Nutrition, Hyderabad, India, acclimatized for 1 week under standard laboratory conditions.

### Immunization and treatment

All groups (except phosphate buffer saline (PBS) control group) were sensitized with CE (10 μg/100 μl PBS) on day 0, 7 & 14 intraperitoneally (*i.p.*). They were challenged with CE (10 μg) or exposed to LPC (1-Palmitoyl-sn-glycero-3-phosphocholine) (Sigma Aldrich) (4.82 μg)^12^ intranasally (*i.n.*) on days 25^th^, 26^th^ and 27^th^ except the CE group, which was exposed to 5% dimethylsulfoxide (DMSO) only, a vehicle control. They were treated 1 hr before challenge, with 5% DMSO (10 μl) or sPLA2 inhibitor LY311727 (Sigma Aldrich) (133 μg/10 μl) intranasally or 24 hr before challenge with anti-CD1d monoclonal antibody (mAb) (50 μg) (clone 1B1, ebioscience, Inc., USA) or isotype (50 μg) intravenously (*i.v.*)^21^,^22^. On day 28^th^ after recording airway hyperresponsiveness (AHR), mice were sacrificed to collect BALF, lungs and spleen. In another experiment mice were exposed on day 0, 7^th^ & 14^th^ to LPC (4.82 μg) or 5%DMSO *i.n.*; on day 15^th^ AHR was recorded and mice were sacrificed to collect the samples.

### Assessment of Airway hyper-responsiveness

Mice were anaesthetized by xylazine (10 mg/Kg) and sodium thiopentone (100 mg/Kg) (*i.p.*). Airway resistance of the respiratory system was recorded using 2, 4, 8, 12, 16, 20, 25 and 30 mg/ ml of methacholine in PBS using Flexi Vent ^TM^ (Scireq, Montreal, Canada) ventilator as described elsewhere^33^.

### Collection of BALF, lungs and spleen

Lungs were lavaged thrice with 0.5 ml PBS^34^. Total collected fluids were spinned and supernatants were stored at −80 °C. Cell pellets resuspended in PBS were used for total leukocyte counting (TLC). A smear of BALF cells was stained with Leishman’s stain to get percentage eosinophils, neutrophils, basophils, plasma cells, lymphocytes, monocytes, macrophages, dendritic cells, and platelets on the basis of cell morphology. The absolute numbers of respective cells were calculated by multiplying with TLC. Lungs were excised out, one part was used for histopathology and another was processed for iROS measurement. Spleen was processed to get splenocytes.

### Measurement of intracellular Reactive Oxygen Species (iROS)

iROS were measured by method described earlier^35^ with some modification. Briefly, lung was homogenized to obtain single cell suspension and iROS were labelled by incubating the cells with 3.3 μM of 2′, 7′-dichlorofluorescein diacetate (DCFH-DA; Molecular Probes, Eugene, OR, USA). Cells were examined under fluorescence activated cell sorter (BD Biosciences, Calibur) and the mean fluorescence intensity of 10, 000 cells were analyzed in each sample and corrected for auto-fluorescence of unlabelled cells.

### Measurement of 8-isoprostanes (8-iso PGF2a)

BALF samples were assayed for 8-isoprostanes using enzyme immunoassay kit (Cayman Chemical Co., MI, USA) following manufacturer’s instructions. The detection limit for 8-isoprostanes was 0.8 pg /ml.

### Measurement of sPLA2 activity

sPLA2 activity was measured in BALF supernatant according to manufacturer’s protocol (Cayman Chemical Co., MI, USA).

### Determination of cytokines by ELISA

Interleukin (IL)-4, IL-5 and IL-13 were measured in BALF using ELISA as per the manufacturer’s instructions (ebioscience, Inc., USA). The detection limit for IL-4, IL-5 and IL-13 was 4.0 pg/ml.

### Histopathology

Lungs were fixed in neutral buffered formalin (10%); embedded in paraffin and 4 μm thick sections were cut using microtome. They were stained with haematoxylin and eosin (H&E) and assessed for inflammation score as described earlier^33^.

### Lipid extraction and LC/MS

LPC 16:0, LPC 18:0 and LPC 18:1 were quantified in BALF by UPLC-MS as described earlier^36^. The samples were spiked with 140 ng/ml of LPC 12:0 (internal standard) and were extracted twice using liquid-liquid extraction (Methanol/Chloroform, 2:1). The mixtures were vortexed, incubated for 15 minutes on ice, followed by addition of 1 ml Chloroform and 1 ml water to separate the phases; vortexed the mixture before spinning at 1,750 × g at 4 °C for 10 minutes. The lower phases were combined; dried using inert gas at RT; dissolved in 100 μl methanol and 10 μl of it was injected into the ESI-MS ion source through auto- sampler of LC system. The system used was Waters APi Quattro micro triple quadrupole mass spectrometer equipped with a Waters UPLC system and a Waters equity C18 column. The detection limit and quantification limit were 9 pg and 30 pg respectively. The standard curves for the LPCs were formed over a range of 1.9–500 ng/ml with linear coefficient of determination (R^2^) = 0.995.

### Flow cytometry to examine NKT cell population

Spleen of each mouse was homogenized to get single cell suspension; spinned at 290 × g, for 10 minutes at 4 °C to get the pellet. RBCs were lysed using RBC lysis buffer. BALF cells and splenocytes were surface stained for LY49 C/I and TCR-β using PE-labelled anti LY49C/I (clone 14B11) and APC- labelled anti TCR-β antibodies (ebioscience, Inc., USA). The cells were acquired using FACSCalibur flow cytometer (BD Biosciences) and analyzed using BD CellQuest Pro software. Dual positive LY49C/I^+^TCR-β^+^ cells were taken as NKT cells.

### Statistical analysis

Data were analyzed using Student t-test to examine differences between control and challenged groups as well as between challenged and treated group. Differences were considered significant at p < 0.05. Data are presented as mean ± SEM for each group.

## Additional Information

**How to cite this article**: Bansal, P. *et al*. Lysophosphatidylcholine plays critical role in allergic airway disease manifestation. *Sci. Rep.* **6**, 27430; doi: 10.1038/srep27430 (2016).

## Supplementary Material

Supplementary Information

## Figures and Tables

**Table 1:** LPC is produced *in vivo* by allergen challenge.

LPC16:0 (ng/ml)	PBS	CE-CE	CE-CE-sPLA2 inhibitor	CE-LPC	CE-LPC-sPLA2 inhibitor
	25 + 1.4	73* + 24	21# + 8.9	118* + 34.2	134 + 0.02

LPC 16:0 was quantified in BALF using LC-MS. Vehicle control: *PBS*; mice sensitized and challenged with cockroach extract: *CE-CE*; pre-administered with sPLA2 inhibitor: *CE-CE-sPLA2 inhibitor*; mice sensitized with cockroach extract and exposed to LPC: *CE-LPC*; pre-administered with sPLA2 inhibitor: *CE-LPC-sPLA2 inhibitor.* Data represent the means ± SEM of values from 4 mice. *p < 0.05 versus *PBS;*^#^p < 0.05 versus *CE-CE*.

**Table 2:** CD1d blockages before CE challenge or LPC exposure decreases Th2 type cytokines.

pg/ml	PBS	CE-CE -PBS	CE-CE-Isotype	CE-CE-mab CD1d	CE-LPC-PBS	CE-LPC-Isotype	CE-LPC-mAb CD1d
IL-13	15 ± 4	58* ± 7	60 ± 25	25# ± 7	60* ± 13	73 ± 32	27$ ± 5
IL-4	10 ± 4	70* ± 21	77 ± 27	20# ± 8	127* ± 59	123 ± 70	36$ ± 6
IL-5	2 ± 0.3	15* ± 1	13 ± 11	1# ± 0.6	15* ± 4	10 ± 2	2$ ± 3

IL-4, IL-5 and IL-13 levels in BALF. Vehicle control: *PBS*; mice sensitized and challenged with cockroach extract and pre-administered with PBS: *CE-CE-PBS*; with isotype: *CE-CE-Isotype;* or with anti-CD1d monoclonal antibody (mAb): *CE-CE-mAb CD1d*; mice sensitized with cockroach extract and exposed to LPC and pre-administered with PBS:*CE-LPC-PBS*; or with isotype: *CE-LPC-Isotype*; or with anti-CD1d mAb: *CE-LPC-mAB CD1d.* Data represent the means ± SEM of values from 4 mice. *p < 0.05 versus *PBS*; ^#^p < 0.05 versus *CE-CE-PBS;*^$^p < 0.05 versus *CE-LPC*-*PBS*.
