# Supplementary material for: Lysophosphatidylcholine plays critical role in allergic airway disease manifestation
Source: Sci Rep. 2016 Jun 10;6:27430. doi: 10.1038/srep27430 (PMC4901285; doi:10.1038/srep27430)
Supplement: Supplementary Information [file srep27430-s1.pdf]

Lysophosphatidylcholine plays critical role in allergic airway disease manifestation

Preeti Bansal [a,b] , Shailendera Nath Gaur[c], Naveen Arora[a*]

aAllergy and Immunology Section, CSIR-Institute of Genomics and Integrative Biology, Delhi.

[b]Department of Biotechnology, University of Pune, Ganeshkhind, Pune 411 007, India

[c]Department of Pulmonary Medicine,V.P.Chest Institue, Delhi University, Delhi.

***Corresponding Address:**

Naveen Arora, Ph.D.

Room 509, Allergy and Immunology Section

CSIR-Institute of Genomics and Integrative Biology,

Delhi University Campus, Mall Road,

Delhi-110007, INDIA

Tel: (011) 27666157, Fax: (011) 27667471

Email: naveen@igib.res.in

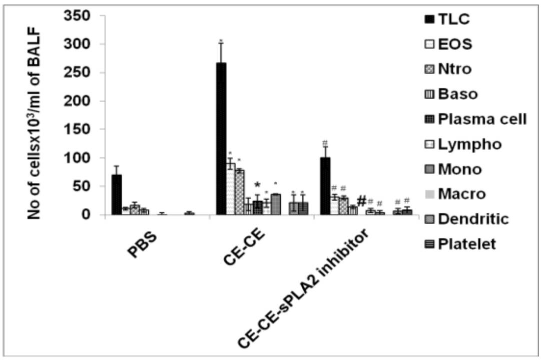

Supplementary Figure1. TLC& DLC were increased in CE challenged mice and reduced on administration of sPLA2 inhibitor. Vehicle control: *PBS*; mice sensitized and challenged with cockroach extract*: CE-CE*; and pre-administered with sPLA2 inhibitor: *CE-CE-sPLA2 inhibitor*. Data represent the means + SEM of values from 4 mice.*, p<0.05 versus *PBS;* #, p<0.05 versus *CE-CE*.

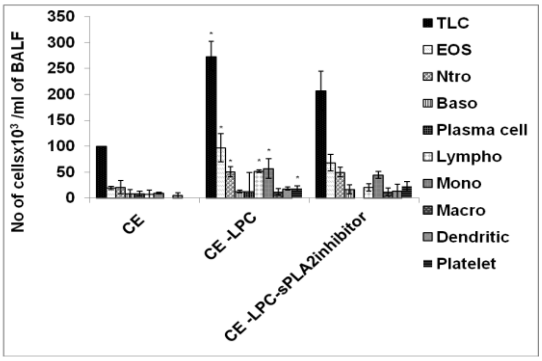

Supplementary Figure2. LPC exposure in CE-sensitized mice increases total and differential cell count in BALF. Mice sensitized with cockroach extract: *CE*; and exposed to LPC: *CE-LPC*; and pre-treated with sPLA2 inhibitor: *CE-LPC-sPLA2inhibitor*. Data represent the means + SEM of values from 4 mice.*, p<0.05 versus *CE*.

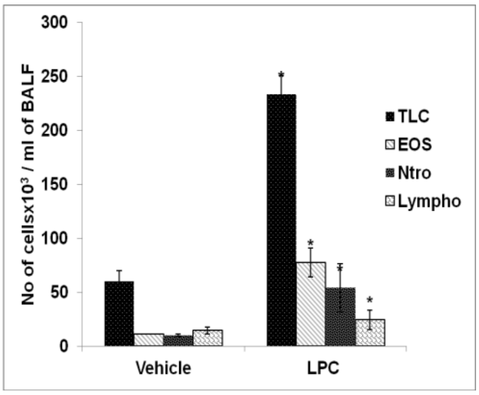

Supplementary Figure3. LPC exposure without CE sensitization increases total and differential cell count in BALF. Mice exposed to 5%DMSO: *Vehicle*; to LPC: *LPC*. Data represent the means $\pm$ SEM of values from 4 mice.*, p<0.05 versus *vehicle*.

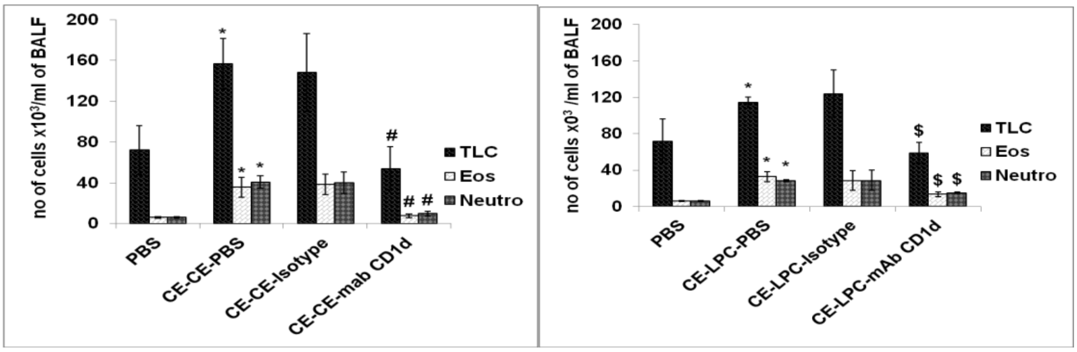

Supplementary Figure4. CD1d blockage before CE challenge or LPC exposure decreases total and differential cell count. a) vehicle control: *PBS*; mice sensitized and challenged with cockroach extract and pre-administered with PBS: *CE-CE-PBS;* with isotype: *CE-CE-Isotype;* or with anti-CD1d monoclonal antibody (mAb): *CE-CE-mAb CD1d*. b) vehicle control: *PBS*; mice sensitized with cockroach extract and exposed to LPC and pre-administered with PBS: *CE-LPC-PBS*; or with isotype: *CE-LPC-Isotype*; or with anti-CD1d mAb: *CE-LPC-mAB CD1d*. Data represent the means $\pm$ SEM of values from 4 mice.*, p<0.05 versus *PBS*; #, p<0.05 versus *CE-CE-PBS* $, p<0.05 versus *CE-LPC-PBS*.
